# Predictors of Recovery in Facial Nerve Palsy: Insights From an Observational Study in Odisha, India

**DOI:** 10.7759/cureus.58949

**Published:** 2024-04-24

**Authors:** Ikhita Misra, Bandana Rath, Ishani Rath, Himel Mondal

**Affiliations:** 1 Otorhinolaryngology, Shri Jagannath Medical College and Hospital, Puri, IND; 2 Pharmacology, Saheed Laxman Nayak Medical College and Hospital, Koraput, IND; 3 Biochemistry, Veer Surendra Sai Institute of Medical Sciences and Research, Burla, IND; 4 Physiology, All India Institute of Medical Sciences, Deoghar, Deoghar, IND

**Keywords:** house-brackmann, electroneurography, india, odisha, bell's palsy, otolaryngology, nerve degeneration, nerve conduction studies, prognosis, facial nerve

## Abstract

Background

Facial nerve paralysis, leading to the loss of facial expression, poses significant discomfort to patients. While most individuals exhibit a favorable response to treatment, a subset experiences enduring facial deformities without clearly defined etiology. This study aimed to identify prognostic factors influencing outcomes and quality of life in facial nerve palsy patients, contributing to enhanced clinical management.

Methods

A prospective observational study was conducted in the Otorhinolaryngology Department of Maharaja Krishna Chandra Gajapati Medical College and Hospital, a tertiary care hospital. We included patients presenting with any clinical variety of facial nerve palsy, irrespective of age and gender. Only moribund and noncompliant cases were excluded. Patients underwent clinical assessment using the House-Brackmann (HB) grading at presentation and were subsequently monitored at three weeks, three months, and six months post-onset to assess recovery.

Results

Out of 66 patients, 18 (27.27%) fully recovered at three weeks, 50 (75.76%) recovered at three months, and 54 (81.82%) at six-month follow-up. Incomplete recovery was observed in 13 (19.69%) patients. Factors associated with favorable outcomes included younger age of onset (p = 0.003), lower baseline HB grade (IV or less) (p = 0.001), Electroneurography Degeneration Index (ENoG DI) of <70% (p < 0.0001), early initiation of treatment (within five days of onset) (p = 0.0003), and absence of comorbid conditions (p = 0.03). Gender and affected side (left or right) did not influence the outcome.

Conclusion

In summary, age, associated comorbid conditions, baseline HB grade, and extent of facial nerve degeneration are crucial predictors of outcomes in facial nerve palsy. This knowledge can guide clinicians in optimizing treatment strategies for improved patient care.

## Introduction

Facial paralysis is a disturbing neurological condition that deprives the patient of voluntary and involuntary facial movement [1]. The condition not only causes functional deficiency, but it can also lead to psychological difficulties and have a dramatic impact on interpersonal relationships [2]. Spontaneous unilateral facial palsy in the absence of an identifiable cause remains the most common cause [3]. In India, the infective cause of facial nerve paralysis remains a threat, especially in rural populations, due to factors like low socioeconomic status, illiteracy, malnutrition, poor hygienic conditions, and overcrowding [4,5]. The majority of the cases of peripheral facial paralysis present as Bell’s palsy. Among the various metabolic conditions causing facial palsy, diabetes, and pregnancy are considered risk factors for Bell’s palsy [5,6].

Interest in understanding the factors that influence the prognosis and recovery of facial nerve palsy is important for proper management of the cases [7,8]. Identifying predictors of recovery is crucial for informing treatment decisions, counseling patients, and optimizing outcomes. Electroneurography (ENoG) is a valuable diagnostic tool used in the assessment and prognosis of facial nerve palsy. ENoG measures the electrical activity of the facial nerve by stimulating it at different points along its course and recording the resulting muscle responses [9].

While several studies have investigated this topic, there is a need for more research, particularly in various regions in India where diverse populations with unique demographic, genetic, and environmental factors may influence recovery patterns. Hence, we conducted this study to identify the prognostic indicators of outcomes in facial palsy in a tertiary care setting in Odisha state, India.

## Materials and methods

Type and setting

This was a prospective observational study carried out in the Department of Otorhinolaryngology at a tertiary care center, the Maharaja Krishna Chandra Gajapati Medical College and Hospital between the years 2017 and 2020. The hospital is situated in the southeastern part of the state of Odisha.

Ethics

The protocol of the study was approved by the Institutional Ethics Committee (approval no 401/2017), and written informed consent was obtained from each patient before commencing the work. All the research participants in the study attained the age of 18 years; hence, they are adults who can consent themselves to voluntary participation.

Participants

We used a hospital-based convenience sample for this study. All the patients presented with any type of facial palsy during the study period were included in the study. Only the moribund and the noncompliant cases were excluded. Regardless of age and gender, all patients aged more than 18 years were included. Any patients who are planned for surgical treatment were excluded from the study.

Data collection

The basic demographic characteristics of all patients were recorded. Socioeconomic status was categorized according to the modified Kuppuswamy scale [10]. The patient’s baseline characteristics were assessed before initiating the treatment. Each patient was clinically assessed for the degree of paresis or paralysis caused by facial palsy using the HB scale system [11]. ENoG was performed on all the patients following standard protocol. ENoG examines the elicited compound muscle action potential of a single facial muscle. The major trunk was stimulated supramaximally as it exited the stylomastoid foramen using bipolar electrodes [12].

All evaluations were made at the patient’s first visit and at three weeks, three months, and six months of follow-up. Medical treatment consisted of steroid monotherapy and antiviral agents or antibiotics. Prednisolone 1 mg/kg/day for five days, followed by a 10-day taper, and oral acyclovir 200-400 mg five times daily for 10 days was advised to all patients.

Statistical analysis

Descriptive statistics was applied to express the proportion or mean and standard deviation. A univariate binary logistic regression test was used to identify the correlates of favorable outcomes. Two-tailed p-values of <0.05 were considered significant. Data were analyzed using the IBM SPSS Statistics for Windows, Version 16 (Released 2007; IBM Corp., Armonk, New York, United States).

## Results

A total of 66 patients (39 male and 27 female) with a mean age of 38.5 ± 8.21 years were observed for six months. The demographics of the patients are described in Table 1.

**Table 1:** Demographic characteristics of the study population SD: Standard deviation Socioeconomic status is according to the modified Kuppuswamy scale [10]

Parameter	Category	Number (%)
Age (mean ± SD)		38.5 ± 8.21
Gender	Male	39 (59.09)
	Female	27 (40.91
Residence	Urban	20 (30.3)
	Rural	46 (69.7)
Socioeconomic status*	Class I	2 (3.03)
	Class II	11 (16.66)
	Class III	19 (28.78)
	Class IV	20 (30.3)
	Class V	14 (21.21)

The patients according to the etiology, side of infection, and baseline HB grade are shown in Table 2.

**Table 2:** Etiology, side of infection, and baseline House-Brackmann grade of the patients HB: House-Brackmann

Parameter	Category	Number (%)
Etiology	Bell’s palsy	43 (65.15)
	Infective	14 (21.2)
	Trauma	7 (10.6)
	Carcinoma	2 (3.03)
Side of infection	Right	44 (66.67)
	Left	22 (33.33)
Baseline HB grade	Grade I	0
	Grade II	16 (24.24)
	Grade III	13 (19.69)
	Grade IV	31 (46.96)
	Grade V	5 (7.57)
	Grade VI	1 (1.51)

**Figure 1:**
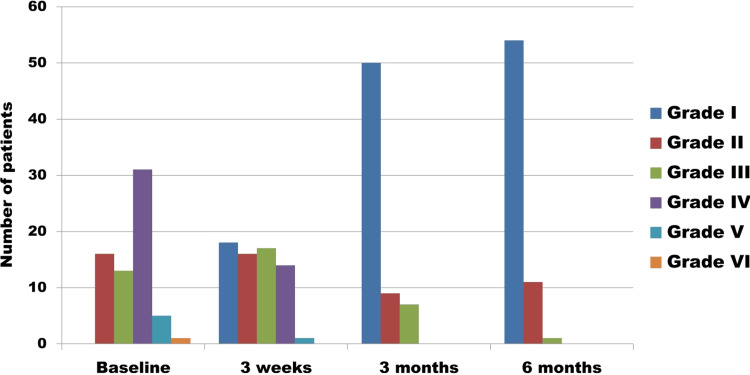
House-Brackmann grading of facial palsy at different times of visit of the study population HB: House-Brackmann

The majority of the patients had Bell’s palsy on the right side with a grade IV HB score. Pre- and posttreatment HB grades of patients at three weeks, three months, and six months of follow-up are shown in Figure 1.

It is evident that 18 (27.27%) patients had recovered completely at three weeks. At three-month follow-up, 50 (75.76%) had turned to HB grade I, and at six-month follow-up, 54 (81.82%) patients had recovered completely. Incomplete recovery was seen in 13 (19.69%) of facial palsy patients even after six months of follow-up.

We observed that among all patients, 41 (62.12 %) patients had ENoG Degeneration Index (ENoG DI) of <70%, 20 (30.3%) had ENoG DI of 71%-90%, and five (7.58%) patients had ENoG DI of >90%.

The correlates of recovery from facial palsy at six months of follow-up are shown in Table 3.

**Table 3:** Correlates of recovery from facial palsy at six months of follow-up HB: House-Brackmann; ENoG DI: Electroneurography Degeneration Index

Parameters	Category	Number	Complete recovery	Partial recovery	Odd ratio	Chi-square	95% confidence interval	p-value
Age (years)	<50	53	47	6	6.7	8.51	1.68-26.76	0.003
	≥50	13	7	6				
Gender	Male	39	30	9	0.41	1.54	0.10-1.71	0.21
	Female	27	24	3				
Basal HB grade	≤IV	60	52	8	13	10.43	2.04-83	0.001
	>IV	6	2	4				
Coexisting disease/condition	yes	9	5	4	0.2	4.83	0.04-0.93	0.03
	no	57	49	8				
ENoG DI	<70	41	36	5	12.8	19.21	3.7-44.32	<0.0001
	>70	25	9	16				
Side of affection	Right	44	37	7	1.56	0.46	0.43-5.61	0.5
	Left	22	17	5				
Onset of treatment (days)	<5	49	45	4	10	12.84	2.47-40.46	0.0003
	>5	17	9	8				

The age, basal HB grade, any coinfection or coexisting chronic disease, ENoG DI, and time of initiation of treatments were the factors predicting the recovery of the patients.

## Discussion

The study finding suggests that several factors play crucial roles in predicting the recovery of facial palsy patients [13]. Age likely influences recovery rates, with younger patients potentially exhibiting better outcomes. The severity of initial facial nerve dysfunction, indicated by basal HB grade, may also impact recovery, with lower grades correlating with improved prognosis. Coinfections or preexisting chronic diseases could complicate recovery processes, potentially delaying or impeding progress. Additionally, ENoG may provide valuable prognostic information. Finally, the timing of treatment initiation likely plays a pivotal role, with earlier interventions likely associated with better recovery rates. These factors collectively highlight the multifactorial nature of facial palsy recovery, underscoring the importance of comprehensive patient evaluation and tailored management strategies [14].

In this study, we found a predominance of males over females. This aligns with the findings of Atolini Junior et al. and Ayala Mejías et al. who similarly reported a male predominance [15,16]. Moreover, individuals residing in rural areas, characterized by poor socioeconomic conditions and unhygienic environments, exhibited a greater susceptibility to infectious diseases leading to facial palsy. The incidence is also found to be associated with socioeconomic status and residence in rural areas by other studies [17,18]. We found that right-sided facial involvement is more frequently encountered, and this finding aligns with previous research findings [19,20]. Conversely, Ayala Mejías et al. reported a preponderance of left-sided facial involvement [16].

At the time of hospital presentation, the baseline HB grading for facial palsy predominantly fell within grade IV, with grade II and grade III cases occurring equally. This distribution closely resembles the findings of Liriano et al. [21]. We observed complete recovery of the majority of the facial palsy cases within six months, as depicted in Figure 1. This finding is consistent with the results of the study by Cha et al. [22]. However, Volk et al. reported complete recovery in a lower percentage of cases at the six-month follow-up [23]. Discordance may be due to patient characteristics and different treatment modalities.

In our study, we found no significant association between the affected side of the face and recovery, consistent with the findings of Park et al. [24]. Early presentation and prompt initiation of treatment within five days of onset were associated with complete recovery in the majority of cases, a pattern also observed by Volk et al. [23]. Moreover, a baseline ENoG DI of less than 70% (indicating a lesser degree of nerve injury) emerged as a strong predictor of complete recovery, aligning with the observations of Chow et al. [25]. Gender showed no association with recovery outcomes in our study, consistent with the findings of Volk et al. [23].

While not a novel study in the broader context, this research represents a significant contribution as the first of its kind conducted in this specific region within a tertiary care hospital setting. By focusing on the local population and healthcare infrastructure, the study provides region-specific insights into the epidemiology, clinical characteristics, and prognostic factors of facial palsy. This localized approach enhances the relevance and applicability of the findings to the healthcare practices and patient populations within the region.

Despite its contributions, this study has several limitations that warrant acknowledgment. Firstly, being a single-center study conducted in a tertiary care hospital, the findings may not be generalizable to other healthcare settings or population groups. Furthermore, the study's sample size and duration may limit the statistical power and generalizability of the results. The absence of long-term follow-up data may preclude a comprehensive assessment of recovery outcomes beyond the six-month mark.

## Conclusions

In conclusion, the common correlates of complete recovery were younger age of patients without comorbidities, lower initial degree of paralysis, and lower degree of nerve injury as evident from ENoG and early presentation and treatment institution. Thus, proper clinical examination, precise diagnosis, and early institution of therapy would bring a quick and complete recovery.
